# Molecular Cloning, Expression Profile and 5′ Regulatory Region Analysis of Two Chemosensory Protein Genes from the Diamondback Moth, *Plutella xylostella*

**DOI:** 10.1673/031.010.14103

**Published:** 2010-09-10

**Authors:** Liang Gong, Guo-Hua Zhong, Mei-Ying Hu, Qian Luo, Zhen-Zhen Ren

**Affiliations:** Key Laboratory of Pesticide and Chemical Biology, Ministry of Education of P.R. China. South China Agricultural University, Guang Zhou, 510642, Guangdong, China

**Keywords:** cDNA cloning, genomic structure, transcription factor recognition site analysis

## Abstract

Chemosensory proteins play an important role in transporting chemical compounds to their receptors on dendrite membranes. In this study, two full-length cDNA codings for chemosensory proteins of *Plutella xylostella* (Lepidoptera: Plutellidae) were obtained by RACE-PCR. *PxylCSP3* and *Pxyl-CSP4*, with GenBank accession numbers ABM92663 and ABM92664, respectively, were cloned and sequenced. The gene sequences both consisted of three exons and two introns. RT-PCR analysis showed that *Pxyl-CSP3 and Pxyl-CSP4* had different expression patterns in the examined developmental stages, but were expressed in all larval stages. Phylogenetic analysis indicated that lepidopteran insects consist of three branches, and *Pxyl-CSP3* and *Pxyl-CSP4* belong to different branches. The 5′regulatory regions of *Pxyl-CSP3* and *Pxyl-CSP4* were isolated and analyzed, and the results consist of not only the core promoter sequences (TATA-box), but also several transcriptional elements (BR-C Z4, Hb, Dfd, CF2-II, etc.). This study provides clues to better understanding the various physiological functions of CSPs in *P. xylostella* and other insects.

## Introduction

In recent years, the diamondback moth, *Plutella xylostella* (Lepidoptera: Plutellidae) has become the most destructive insect of cruciferous plants throughout the world, and the annual cost for its management is estimated to be US $1 billion (Talekar 1993). In order to find the solution, differentially expressed genes from this insect should be identified, cloned, and studied. In this regard, the study of chemosensory proteins of *P. xylostella* will be helpful in providing critical information about their behavioral characteristics and relative physiological processes.

Insect chemosensory proteins (CSPs) and odorant-binding proteins (OBPs) are believed to be involved in chemical communication and perception, and these two soluble proteins belong to different classes. OBPs have the size of approximately 150 amino acid residues, out of which six highly conserved cysteines are paired to form three disulfide bridges. It has been experimentally demonstrated that OBPs are involved in the binding of pheromones and odorant molecules (Vogt 1881; Kruse 2003; Andronopoulou 2006). CSPs are small proteins of about 110 amino acids that contain four cysteines forming two disulfide bridges (McKenna 1994; Pikielny 1994; Jansen 2007). In comparison to OBPs, which are specifically reported in olfactory sensilla (Vogt and Riddiford 1981; Steinbrecht 1998), the CSPs are expressed more extensively in various insect tissues such as the antennae, head, thorax, legs, wings, epithelium, testes, ovaries, pheromone glands, wing disks, and compound eyes, suggesting that CSPs are crucial for multiple physiological functions of insects (Gong 2007). Similarly, the study of gene expression in different insect stages can reveal the possible extent of activity of these specific genes in the physiology of the different stages.

In the last two decades, insect chemosensory proteins have been studied extensively for their structural properties, various physiological functions, affinity to small molecular ligands, expression pattern in insects, and subcellular localization, but little research has been reported on the analysis of the 5′-regulatory sequence of the chemosensory protein gene. In this study, the full-length cDNA was cloned for two chemosensory protein genes (*Pxyl-CSP3* and *Pxyl-CSP4*) in *P. xylostella*, using rapid amplification of cDNA ends (RACE). It was followed by the genome walking method to obtain the 5′-upstream regulatory sequence of *Pxyl-CSP3* and *Pxyl-CSP4*. The results revealed not only the core promoter sequences (TATA-box), but also several transcriptional elements (BR-C Z4, Hb, Dfd, CF2-II etc).

## Materials and Methods

### Insects

*P. xylostella* pupae were collected from an insecticide-free cabbage field and taken to the laboratory for rearing. Larvae were allowed to feed on cabbage leaves in the insect growth room with conditions set at 25 ± 1° C, 16:8 L:D, and 70–85% RH until pupation.

### RNA preparation and synthesis of firststrand cDNA

Total RNA was extracted from adults of *P. xylostella* using the Trizol reagent (Invitrogen, www.invitrogen.com) according the protocol provided by the manufacturer. First-strand cDNA was synthesized from the total RNA with reverse transcriptase AMV and oligod (T)18 (TaKaRa, www.takara-bio.com). 5′- and 3′-RACE-ready cDNA were prepared according to the instructions of the Gene Racer™ Kit protocol (Catalog #: L1500-01, Invitrogen).

### Cloning *of Pxyl-CSP3* and *Pxyl-CSP4*

Two degenerate primers were designed by alignment of published CSP-like transcripts from distantly related species. The 3′ RACE forward primers of *Pxyl-CSP3* and *Pxyl-CSP4* are 5′-(C/T)AC(A/G)GA(T/C)AA(A/G)CA (C/G)GAA(A/G)C(C/A)(A/T)GCCGTGA-3′ and 5′-GAA(A/G)ACCA(C/T)C(C/T)GCGG CAA (G/C/A)TGCA -3′, respectively, and oligod (T)18 was used as the reverse primer. The PCR reaction was performed with the following conditions: one cycle (94° C, 2 min); 35 cycles (94° C, 1 min; 55° C, 1 min; 72° C, 1 min); and a last cycle 72° C for 10 min. The PCR product was then cloned into a pMD-20-T vector (TaKaRa), and positive clones were sequenced.

According to the CSP-like transcript fragment amplified from *P. xylostella* by 3′ RACE degenerate primers, the 5′-RACE specific nest primers were designed and used to amplify the full-length cDNA of *Pxyl-CSP3* and *Pxyl-CSP4.* The 5′-RACE primer and 5′-RACE nest primer of *Pxyl-CSP3* are 5′-CCTCC ACTCCGCGGGCTTGTGGTTGAT-3′ and 5′-TACGCCTTGACAGCGCGCAGTTGGT CC-3′, respectively. The 5′-RACE primer and 5′-RACE nest primer of *Pxyl-CSP4* are 5′-CTTGGCGAAGGAGTCCTTGTACTCTCC-3′ and 5′-TCAGAAGATGTCATCTAAGT TC-3′, respectively. The first PCR conditions were as follows: one cycle (94° C, 2 min); 5 cycles (94° C, 30 s; 72° C, 1 min); 5 cycles (94° C, 30 s; 72° C, 1 min); 25cycles (94° C, 30 s; 66° C, 30 s; 70° C, 1 min); and a last cycle of 72° C for 10 min. Full-length cDNA of *Pxyl-CSP3* and *Pxyl-CSP4* was obtained by overlapping the two cDNA fragments.

**Table 1.**
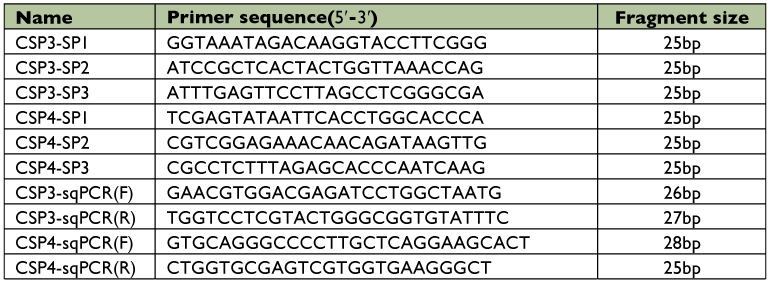
Specific primers (SP1, SP2, and SP3) were designed for (first, second, and third round) genome walking, respectively, which is based on the genome sequence of Pxyl-CSP3 and Pxyl-CSP4. Primers of CSP3-sqPCR and CSP4-sqPCR were designed for RT-PCR.

### Genomic DNA isolation and DNA sequence amplification

Genomic DNA was extracted from *P. xylostella* according to the instructions from the TIANamp Genomic DNA kit protocol (Tiangen, www.tiangen.com). Genomic DNA was precipitated with ddH_2_O, and agarose gel electrophoresis was carried out to determine its quality. It was shown on a single band. The specific primers were designed to amplify the genomic DNA corresponding to the cDNA code region of *Pxyl-CSP3* and *Pxyl-csp4.* In order to clone the genomic sequence of *Pxyl-CSP3*, the sense primer was 5′-ATGAACTCCTTGGTACTAGTATGCCTTG-3′, and the antisense primer was 5′-TACGCCTTGACAGCGCGCAGTTGGTCC-3′. For *Pxyl-CSP4*, the sense primer was 5′-ATGCAGACCGTGACTCTCCTATGCCTG T-3′, and the antisense primer was 5′-TTAATCAGATCCTTCGAGGAACTTGGC G-3′. The PCR reaction was performed with the following conditions: one cycle (94° C, 2 min); 35 cycles (94° C, 30 s; 68° C, 45 s; 72° C, 1 min) and a last cycle 72° C for 10 min. The amplified DNA was sequenced.

### Isolation of genomic 5′- upstream region of *Pxyl-CSP3* and *Pxyl-CSP4*

Genomic DNA of *P. xylostella* was prepared as above. In order to obtain the 5′-upstream regulatory sequences of the chemosensory protein genes, the genome walking approach was performed according to the introductions of the kit (TaKaRa). The PCR principle of the genome walking approach is thermal asymmetric interlaced PCR (Tail-PCR). The specific reverse primers were designed according to 5′-terminal nucleotide sequence of *Pxyl-CSP3* and *Pxyl-CSP4* (Table 1), and the forward primers were supported by the kit. The conditions for the were PCR reaction were set according to the kit's introductions. The PCR fragments obtained through the genome walking approach were detected using 1.5% agarose gel electrophoresis and purified for sequencing using SP3 specific primer.

### RT- PCR analysis

RT-PCR was used to measure gene expression at different developmental stages. The cDNA samples from male and female adults, from all stages of larvae and from pre-pupae and pupae, were prepared using the plant RNA kit (Catalog #: R6827, Omega, www.omega.com) and reverse transcriptase AMV (TaKaRa).

The gene-specific primer was designed from the cDNA sequences of *Pxyl-CSP3* and *Pxyl-CSP4*, named CSP3-sqPCR and CSP4-sqPCR, respectively (Table 1). The 18S rRNA gene of *P. xylostella* was used as the reference with the following primers: 18S-F: 5′-CCGATTGAATGATTTAGTGAGGTCTT-3′; 18S-R: 5′-TCCCCTACGGA AACCTTGTTACGACTT-3′. The cDNA (1–2 µl) was used for amplification, and the final volume of the reaction mixture was 50 µl. The PCR amplification was performed using the following thermal cycle conditions: one cycle (94° C, 2min); 27 cycles (94° C, 30 s; 60° C, 45 s; 72° C,1 min) and a last cycle 72° C for 10 min. PCR products were detected by 1.5% agarose gel electrophoresis.

### Bioinformatics analysis

Amino acid sequences of CSPs (*n* = 27) were retrieved from an NCBI protein search using the keywords “chemosensory protein” and “lepidopteran”. Molecular mass and isoelectric point was predicted using the software, ExPASy (http://www.expasy.ch/). Multiple sequence alignment was carried out with the online service at http://bioinfo.genotoul.fr/multalin/multalin.html (Corpet 1988). Promoter prediction and characterization were carried out using the Neural Network Promoter Prediction (NNPP) server (http://www.fruitfly.org/seq_tools/promoter.html) (Reese 2001). Sequence analysis seeking transcriptional regulation response elements was carried out with TFSEARCH (http://www.cbrc.jp/research/db/TFSEARCH.html) (Heinemeyer 1998). The signal peptide was predicted using SignalP 3.0 (Nielsen 1997) at http://www.cbs.dtu.dk/services/SignalP-3.0/. The phylogenetic tree was constructed using MEGA 3.0 software (Kumar 2004) using the neighbour joining method, and it was reconstructed with 1000-replicate bootstrap analysis.

## Results

### Gene cloning of *Pxyl-CSP3* and *Pxyl-CSP4*

**Figure 1.**
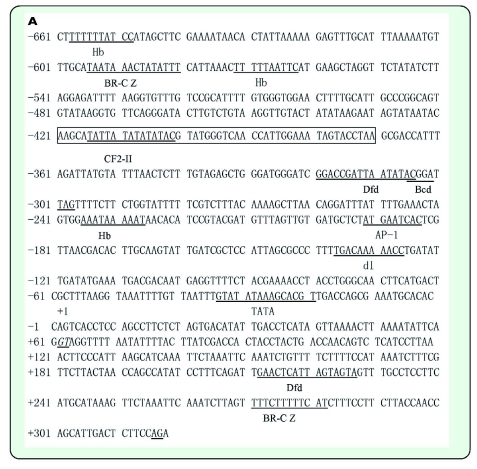
Part of the nucleotide sequence of the *Pxyl-CSP3* 5′upstream region (A) and cDNA and predicted amino acid sequence of *Pxyl-CSP* (B). A: Putative promoter sequences are indicted in box, several transcriptional elements are shown by underline. Transcriptional start site is designated as +1 and the 5′ splice site “GT” and 3' splice site “AG” of this intron are shown by italic and underlined fonts. B: Conserved Cys sites are shaded in gray.The signal peptide is boxed. The locations of intron are shown by boldfaced minuscule letters in bracket. The stop codon is indicated by an asterisk. The locations of the initial degenerate primers for 3′RACE are represented by arrow. The start codon and AATAAA-box showed in boldface.

A 526 bp cDNA of *Pxyl-CSP3* (Figure 1B) was obtained by RACE-PCR using the degenerate primers. The cDNA included a 62 bp 5′ untranslated region (UTR), a 108 bp 3′ UTR, with an AATAAA box and 25 bp poly (A) tail, and a 381 bp open reading frame (ORF) that encodes 126 amino acids. It exhibited significant similarity to CSP5 of *Bombyx mori* (59%), CSP3 of *Bombyx mandarina* (58%), and CSP3 of *Mamestra brassicae* (58%), as revealed by Blast database research. The deduced protein has a computed molecular mass of 14.1 kDa and a predicted isoelectric point of 8.79.

**Figure 2.**
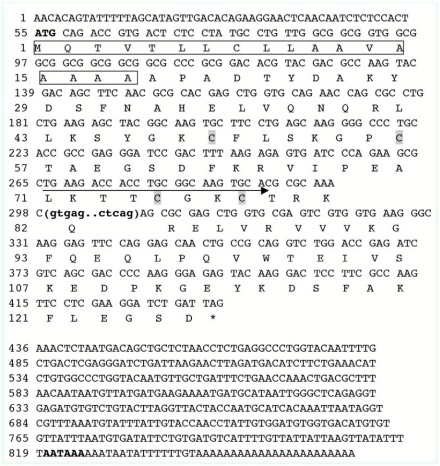
cDNA and predicted amino acid sequence of Pxyl-CSP4 gene. Conserved Cys sites are shaded in gray. The signal peptide is boxed. The locations of intron are shown by boldfaced minuscule letters in bracket and the introne sequence are shown in the following. The stop codon is indicated by an asterisk. The locations of the initial degenerate primers for 3′RACE are also shown. The start codon and AATAAA-box are shown in boldface. High quality figures are available online.

An 864 bp cDNA of *Pxyl-CSP4* (Figure 2) was obtained by RACE-PCR using the degenerate primers. The cDNA included a 54 bp 5′ untranslated region (UTR), a 429 bp 3′ UTR, with an AATAAA box and 23 bp poly (A) tail, and a 381 bp open reading frame (ORF) that encodes 126 amino acids. It exhibited significant similarity to CSP6 of *Papilio xuthus* (68%), CSP8 of *Bombyx mori* (52%) and CSP4 of *Choristoneura fumiferana* (46%), as revealed by Blast database research. The deduced protein has a computed molecular mass of 14.0 kDa and a predicted isoelectric point of 8.25.

### Genomic characterization of *Pxyl-CSP3* and *Pxyl-CSP4*

PCR amplification of genomic DNA with primers designed corresponding to the cDNA of *Pxyl-CSP3* and *Pxyl-CSP4* resulted in products of about 1452 bp and 1268 bp, respectively. By comparing their genomic sequence and cDNA sequence, it was found that *Pxyl-CSP3* and *Pxyl-CSP4* included one intron, and the intron began with ‘GT’, ended with AG, and had 926 bp and 404 bp, respectively. The sequences of the exon/intron-splicing junctions of *Pxyl-CSP3* and *Pxyl-CSP4* are shown in Figure 1B and Figure 2, respectively.

### 5′ upstream regulatory region analysis of *Pxyl-CSP3* and *Pxyl-CSP4*

Using the genome working approach, the 5′ regulatory regions of *Pxyl-CSP3* and *Pxyl-CSP4* were isolated and had 2242 bp and 533 bp, with the Genebank Numbers FJ948816 and FJ948817, respectively. Nucleotide sequence alignment of the isolated genomic sequence with the full-length *Pxyl-CSP4* cDNA showed that the nucleotide sequence of 264 bp was isolated from the 5′ UTR of *Pxyl-CSP4*, including a part of the intron sequence.

Nucleotide sequence alignment of the isolated genomic clone with the full-length *Pxyl-CSP3* cDNA revealed that the 5′ UTR (Figure 1A) was interrupted by an intron of 323 bp, and thus was split in two exons of 61 and 75 bp, respectively. This intron also is in line with the GT-AG rule. The *Pxyl-CSP3* 5′ upstream region of 1921 bp was analyzed to predict the transcription factor binding site, using the online server of TFSEARCH. The results of Figure 1A showed that the 5′ upstream region of *Pxyl-CSP3* included not only the core promoter sequences (TATA-box), but also several transcriptional elements (BR-C Z4, Hb, Dfd, CF2-II, etc.).

### Expression profile of *Pxyl-CSP3* and *Pxyl-CSP4*

**Figure 3.**
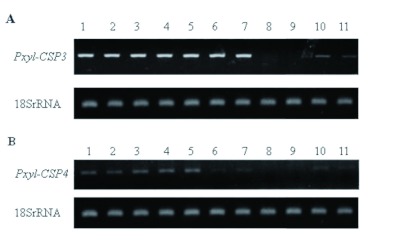
RT-PCR analysis showed the expression of *Pxyl-CSP3* (A) and *Pxyl-CSP4* (B) at different developmental stages. The horizontal data represents from 1 to 11: first instar larva, second instar larva, third instar larva, fourth instar larva ♀, fourth instar larva ♂, pre-pupa ♀, pre-pupa ♂, pupa ♀, pupa ♂, adult ♀, and adult ♂. High quality figures are available online.

RT-PCR was used to investigate the expression at different developmental stages. The results showed that *Pxyl-CSP3* and *Pxyl-CSP4* have different expression patterns in examined developmental stages. *Pxyl-CSP3* (Figure 3A) was highly expressed in the first instar larva, second instar larva, third instar larva, fourth instar larva ♀, fifth instar larva ♂, pre-pupa ♀, and pre-pupa ♂, but no expression was obtained in pupa ♀ or pupa ♂. Lower expression was observed in adult ♀ and adult ♂. In the case of *Pxyl-CSP4* (Figure 3B), higher expression was found in first instar larva, second instar larva, third instar larva, fourth instar larva ♀, and fifth instar larva ♂, while pre-pupa ♀, pre-pupa ♂, adult ♂, and adult ♂ expressed lower expression, and no expression was found in pupa ♀ or pupa ♂.

**Figure:**
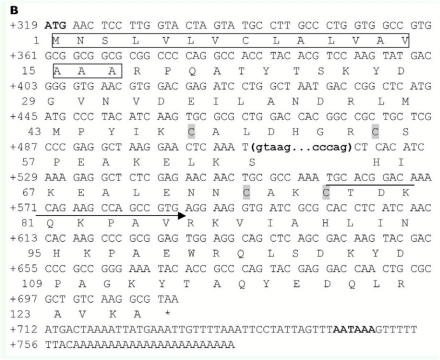

### Homology and phylogenetic analysis

**Figure 4.**
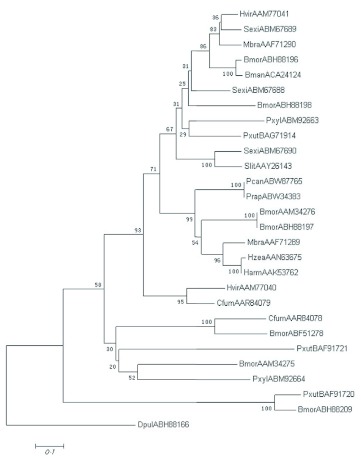
A phylogenetic tree of CSPs from 2 *Plutella xylostella* CSPs and 25 lepidopteran insect homologs. Protein Ids are indicated. Abbreviations: Pcan, *Pieris conidia*; Prap, *Pieris rapae*; Hzea, *Helicoverpa zea;* Harm, *Helicoverpa armigera;* Pxut, *Papilio xuthus;* Mbra, *Mamestra brassicae;* Hvir, *Heliothis virescens;* Bmor, *Bombyx mori;* Cfum, *Choristoneura fumiferana;* Sexi, *Spodoptera exigua;* Slit, *Spodoptera litura;* Dpul, *Daphnia pulex.* High quality figures are available online.

The evolutionary relationships among the two *P. xylostella* CSPs and 25 lepidopteran insect homologs that are reported so far were investigated. An unrooted neighbor-joining tree (Figure 4) was constructed to represent the relationship among selected CSPs. One CSP of *Daphnia pulex* was used for the outgroup. The results obtained from the phylogenetic analysis showed that lepidopteran insects consist of three branches, and *Pxyl-CSP3* and *Pxyl-CSP4* belong to different branches as well. It provides clues about the diversification of these proteins in this insect order.

Amino acid sequence alignment from selected lepidopteran CSPs revealed that the conserved Cys spacing pattern was CX6CX18CX2C, and it was the common spacing pattern within the CSP family. *Pxyl-CSP3* and *Pxyl-CSP4* have only 38% similarity. *Pxyl-CSP3* showed high similarity to CSP3 of *Mamestra brassicae* (56%), but *Pxyl-CSP4* showed higher similarity to CSP of *Papilio xuthus* (69%), suggesting that CSPs from the species of *P. xylostella* are more similar to CSPs from other species than to that of some members of its own.

## Discussion

Insect chemosensory proteins (CSPs) have been supposed to transport chemical stimuli from air to olfactory receptors. However, CSPs are expressed in various insect tissues including non-sensory tissues, suggesting that these proteins are also vital for other physiological processes. In this study, two full-length cDNA coding for chemosensory proteins of *P. xylostella* (Pxyl-CSP3 and Pxyl-CSP4) were obtained by RACE-PCR, and the GenBank accession numbers are ABM92663 and ABM92664, respectively.

**Figure 5.**
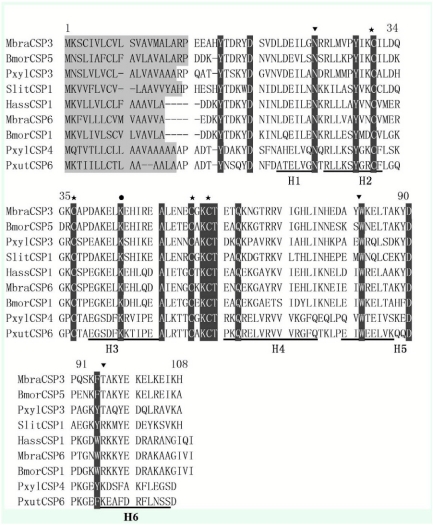
Deduced amino acid alignment of *Plutella xylostella* and CSPs from other insects. Signal peptides are indicated by background of grey and conserved cysteines residues are marked by star, conserved lysine (Lys) showed by dark cycle. Alpha helical domains (H1–H6) identified according to *Mamestra brassicae* chemosensory protein 6. Aromatic residues at positions 27, 81, and 94 are shown by dark arrowheads. High quality figures are available online.

The majority of CSP genes in insects have an intron; only three *Anopheles gambiae* and four *Drosophila* CSP genes lack introns; the intron splice site is always located on one nucleotide after a conserved lysine (Lys) codon, and its position is indicated by dark cycle (Figure 5). These results are accordant with the findings of Wanner (2004), as the intron splice sites of *Pxyl-CSP3* and *Pxyl-CSP4* are after the nucleotide acids AAA (Lys) T and AAA (Lys) C, respectively. This conserved splice site is considered to be a general characteristic of the CSP gene family, so it is evident that these clones belong to this family.

Insect CSP genes are not only expressed in the olfactory tissues but also in non-olfactory tissues, including the antennae, head, thorax, legs, wings, epithelium, testes, ovaries, and pheromone glands (Gong 2007; Lu 2007).

This wide tissue expression pattern may indicate that CSPs have olfactory and nonolfactory functions. The data here shows that *Pxyl-CSP3* and *Pxyl-CSP4* have different expression profiles in different developmental stages and that they were all expressed in larval stage. So, it is suggested that *Pxyl-CSP3* and *Pxyl-CSP4* have important functions for early development of *P. xylostella*, but the detailed physiological role is still unknown.

CSPs are widely distributed in insect species and so far have been identified in 10 insect orders, including Lepidoptera (Maleszka 1997; Robertson 1999; Nagnan-L Meillour 2000; Picimbon 2000), Diptera (McKenna 1994; Pikielnyl 1994), Hymenoptera (Danty 1998; Briand 2002), Orthoptera (Angeli 1999), Phasmatodea (Tuccini 1996), Blattoidea (Kitabayashi 1998), Hemiptera (Jacobs 2005), Phthiraptera (Zhou 2006), Trichoptera (Zhou 2006), and Coleoptera (Zhou 2006). A CSP-like protein has been reported in a non-insect arthropod, the brine shrimp *Artemia franciscana*, suggesting that CSPs might be present across the arthropods (Pelosi 2006). But CSPs belong to a conserved protein family, and CSPs in different insect orders have shared common characteristics such as: conserved Cys residues spacing pattern; aromatic residues at positions 27, 85, and 98 that are also highly conserved; and a novel type of α-helical structure with six helices connected by α-α loops. This data (Figure 5) corresponds to those sequence and structure characteristics as confirmed by multiple sequence alignment. Homology and phylogenetic tree analysis indicated that CSPs from the species of *P. xylostella* are more similar to CSPs from other species than to some members of its own, suggesting evolutionary divergence in CSPs of *P. xylostella.*

Gene promoter sequence and transcription factor recognition site analysis are important for understanding regulation and feedback mechanisms in specific physiological processes. This study succeeded in isolating the 5′ regulatory region of *Pxyl-CSP3* and is the first report about the 5′ upstream regulatory sequence of the insect chemosensory protein gene. This data revealed that the 5′ regulatory region of *Pxyl-CSP3* have a lot of specific transcription factor binding sites including BR-C Z4, Hb, Dfd, CF2-II, etc. The transcription factor binding site of BR-C Z4 has appeared many times in this regulatory region, which may play an important role for duplication and expression of *Pxyl-CSP3.* It has been reported that BR-C Z4 directly mediates the formation of the steroid hormone ecdysone for *Drosophila* melanogaster larvae metamorphosis (Kalm 1994). However, there is no direct evidence for the role of CSPs in insect metamorphosis, but some scientists reported that CSPs are expressed in the pheromonal gland of *M. brassicae* and the ejaculatory duct of *D. melanogaster* (Jacquin-Joly 2001; Sabatier 2003). A recent report also showed that the CSP homologue of *Agrotis segetum* has upregulation expression in the insect-pheromone binding domain; this CSP has also been reported to be the same as juvenile hormone binding protein (Strandh 2008). These findings are in line with the data from the transcription factor binding site analysis, as well as the high expression in the larval stage, which may implicate a function of *Pxyl-CSP3* for steroid hormone production or transport in this insect larval stage. Chemosensory protein association with insect development has been confirmed by many scientists, especially in embryo development. For example, CSP5 of *Apis mellifera* is an ectodermal gene involved in embryonic integument formation (Maleszka 2007). In the cockroach *Periplaneta americana*, the CSP p10 increases transiently during limb regeneration at the larval stages (Kitabayashi 1998). The transcription factor binding sites of Hb, Dfd, and CF2-II have been shown to be involved in developmental regulation; for instance, Hb regulates gene expression in the development of the thoracic region of Drosophila embryos (McGregorl 2001), and CF2 may potentially regulate distinct sets of target genes during development (Gogos 1992). This study will provide clues to better understand the function of CSPs in insect development.

## Abbreviations

BR-C Z4: Broad-Complex Z4;
CSP: Chemosensory protein;
CF2-II: Zinc finger domain;
Dfd: Deformed;
Hb: Hunchback;
ORF: open reading frames;
Pxyl: *Plutella xylostella;*
RACE: rapid amplification of cDNA ends;
UTR: untranslated region.
